# Differences in Spontaneous Intracerebral Hemorrhage Cases between Urban and Rural Regions of Taiwan: Big Data Analytics of Government Open Data

**DOI:** 10.3390/ijerph14121548

**Published:** 2017-12-10

**Authors:** Hsien-Wei Ting, Ting-Ying Chien, K. Robert Lai, Ren-Hao Pan, Kuan-Hsien Wu, Jun-Min Chen, Chien-Lung Chan

**Affiliations:** 1 Department of Information Management, Yuan Ze University, Tao-Yuan 320, Taiwan; ting.ns@gmail.com; 2 Department of Neurosurgery, Taipei Hospital, New Taipei City 242, Taiwan; 3 Innovation Center for Big Data and Digital Convergence, Yuan Ze University, Tao-Yuan 320, Taiwan; tinin@saturn.yzu.edu.tw; 4 Department of Computer Science and Engineering, Yuan Ze University, Tao-Yuan 320, Taiwan; krlai@saturn.yzu.edu.tw (K.R.L.); pan@51donate.com (R.-H.P.); miranda84315@gmail.com (K.-H.W.); w2301231@gmail.com (J.-M.C.); 5 Department of Information Management, Tunghai University, Taichung 407, Taiwan

**Keywords:** health care accessibility, medical expenditure

## Abstract

This study evaluated the differences in spontaneous intracerebral hemorrhage (sICH) between rural and urban areas of Taiwan with big data analysis. We used big data analytics and visualization tools to examine government open data, which included the residents’ health medical administrative data, economic status, educational status, and relevant information. The study subjects included sICH patients of Taipei region (29,741 cases) and Eastern Taiwan (4565 cases). The incidence of sICH per 100,000 population per year in Eastern Taiwan (71.3 cases) was significantly higher than that of the Taipei region (42.3 cases). The mean coverage area per hospital in Eastern Taiwan (452.4 km^2^) was significantly larger than the Taipei region (24 km^2^). The residents educational level in the Taipei region was significantly higher than that in Eastern Taiwan. The mean hospital length of stay in the Taipei region (17.9 days) was significantly greater than that in Eastern Taiwan (16.3 days) (*p* < 0.001). There were no significant differences in other medical profiles between two areas. Distance and educational barriers were two possible reasons for the higher incidence of sICH in the rural area of Eastern Taiwan. Further studies are necessary in order to understand these phenomena in greater depth.

## 1. Introduction

Spontaneous intracerebral hemorrhage (sICH) incurs a high medical expenditure, especially emergency and intensive care facilities [1,2]. Meanwhile, many sICH patients have multiple chronic conditions [2,3]. Both hypertension and diabetes mellitus are found to be the most frequent comorbidities of the sICH patients before being attacked [2,3]. Continuity of care for the patients with the comorbidities will decrease the risk of sICH [3]. It is the key performance index for the quality of care for chronic diseases. Disparity in health care facilities between rural areas and urban areas is huge [4,5,6]. There are significantly different incidences in some diseases and public health problems in rural area due to different health care accessibilities. That the rural areas have fewer health care facilities and this fact will influence the outcome of health care [6]. Socioeconomic status and educational status also influence health care accessibility and the outcome of health care [5,7,8]. Stroke patients in China of a lower income status, lower educational status and rural location were found to have higher mortality rates [7]. Thus, it is important to analyze the different status in sICH between rural and urban regions with big data.

The concept of big data has been defined as four Vs: Large amount of data (volume), diversity of the structure of the data (variety), quick data access and data management (velocity), and the quality of being true and real-world data (veracity) [9]. Many researchers used administrative data related to health care [1]. However, few researchers have integrated these data with other government open data. This study integrated Taiwan National Health Insurance data [10,11,12] which covered 99% of Taiwan’s population (about 23 million residents) and government open data which including household registration database of the Department of Household Registration (http://www.ris.gov.tw/en/web/ris3-english/home) [13], the 2010 population and housing census (http://ebas1.ebas.gov.tw/phc2010/chinese/rchome.htm) [14], smoker statistics data and Taiwan Geographic Information Systems (GIS) data (https://data.gov.tw/) [15] and using big data analytics and visualization tools to evaluate the status of sICH in rural and urban areas of Taiwan.

## 2. Materials and Methods 

### 2.1. Data Source

This study integrated the National Health Insurance Research Database (NHIRD), the household registration database of the Department of Household Registration, the 2010 population and housing census, and the Government Open Data Platform in Taiwan with big data analytics systems using the platform of the Innovation Center for Big Data and Digital Convergence.

### 2.2. Data Protection and Permission

The personal information of all subjects was encrypted using a double scrambling protocol for research purposes to protect patient privacy. All researchers who wish to use the NHIRD and its data subsets are required to sign a written agreement declaring that they have no intention of obtaining information that could potentially violate the privacy of patients or care providers. This study was approved by the Institutional Review Board (IRB) of the Taipei Hospital (IRB Approval Number: TH-IRB-0015-0003), and the protocol was evaluated by the National Health Research Institutes (NHRI), which consented to this planned analysis of the NHIRD (Agreement Number: NHIRD-104-183).

### 2.3. Data Management

**Figure 1:**
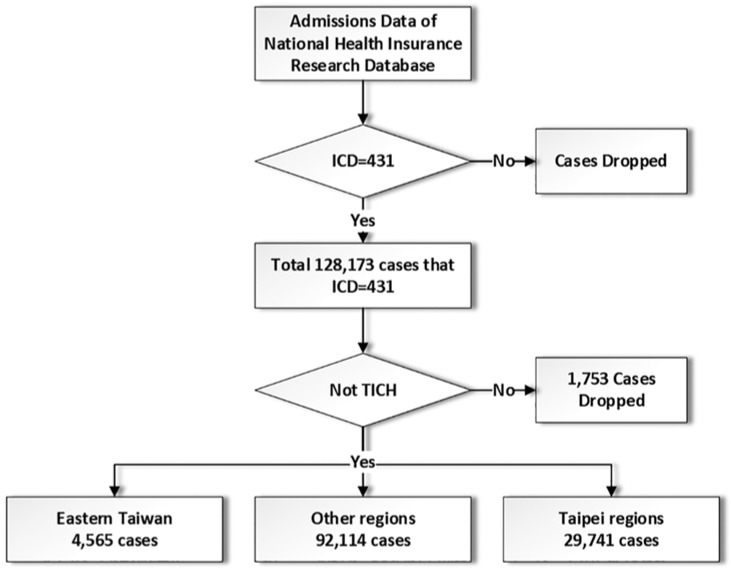
Flow chart of management of data from the National Health Insurance Research Database in Taiwan. * TICH: Traumatic Intracerebral Hemorrhage.

The inclusion criterion in this study was patients with first-attack sICH, identified by a principal diagnosis code in the International Classification of Diseases 9th version (ICD-9) of 431. There were 128,173 sICH cases registered from 2001 to 2011. Patients admitted due to traumatic intracranial hemorrhage (TICH) (ICD-9 codes: 800 to 804.99, 850 to 854.19, 959.01, and 959.09) were excluded [2], resulting in 1753 cases being excluded from this study. The Taipei region (Taipei City and New Taipei City, 29,741 cases) and Eastern Taiwan (Taitung and Hualian, 4565 cases) were included in this study (Figure 1).

Information on patients’ profiles and hospital admission expenditures were collected. A prolonged ICU stay of sICH patients was defined as admission to ICU for more than 10 days [1]. The Charlson Comorbidity Index (CCI) was used to evaluate the severity of a patient’s condition [16,17].

### 2.4. Statistics and Data Analysis

The Student *t-*test was used to analyze the continuous data, and the χ^2^ test was used for the categorical data. Statistical analysis was conducted using SPSS version 12.0 (SPSS Inc., Chicago, IL, USA). The big data analytics and visualization tools were constructed at the Innovation Center for Big Data and Digital Convergence, Yuan Ze University. Statistical significant was set at *p* < 0.05.

## 3. Results

**Figure 2:**
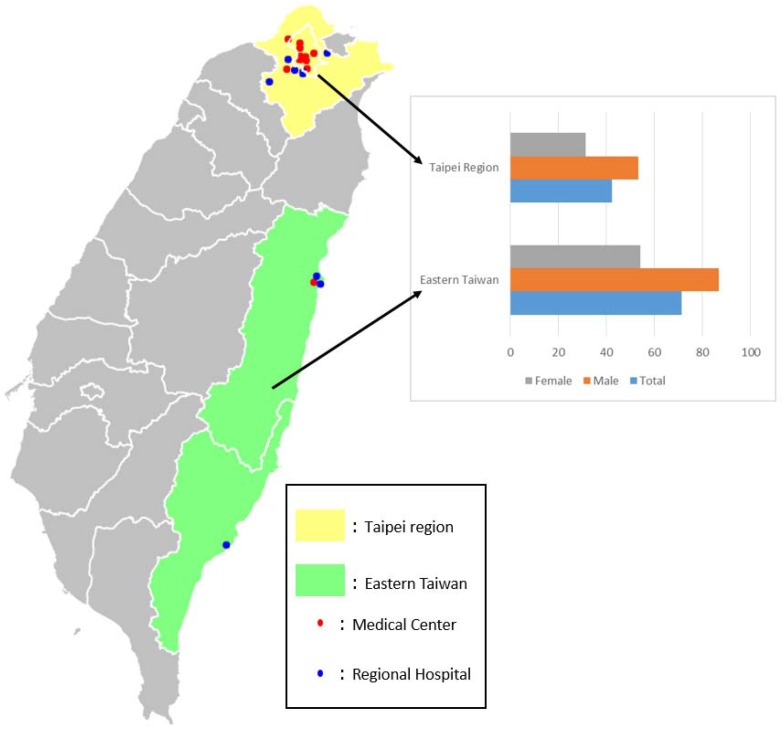
Map shows the different regions of Taiwan discussed in this study. The yellow area: Taipei region; The green area: Eastern Taiwan. Red circle: Medical center; blue circle: Regional hospital.

Taipei City and New Taipei City (Taipei region) are urban areas in Taiwan. Taitung and Hualian (Eastern Taiwan) are rural areas in Taiwan (Figure 2). The low-income resident proportion in Eastern Taiwan (3.1%) was twice that in the Taipei region (1.3%). Regarding educational status, the proportion of residents with a university/college level of education in Eastern Taiwan (25.5%) was significantly lower than that in the Taipei region (46.7%) (Table 1). The mean coverage area per hospital in Eastern Taiwan (452.4 km^2^) was significantly larger than that in the Taipei region (24 km^2^) (*p* < 0.001). The total numbers of beds and ICU beds in the Taipei region (33,570 beds/1861 ICU beds) were significantly greater than those in Eastern Taiwan (5588 beds/238 ICU beds) (*p* < 0.001). The same was true for the medical staffing level: The numbers of medical staff and physicians in the Taipei region (44,556/8529 persons) were also significantly higher than in Eastern Taiwan (4555/733 persons) (*p* < 0.001) (Table 2).

If discussing permanent residents only, there were further differences between Eastern Taiwan and the Taipei region. The numbers of hospital beds/ICU beds per 10,000 population in Eastern Taiwan (109.4/4.7 beds) were significantly greater than those in the Taipei region (50/2.8 beds) (*p* < 0.001). Interestingly, the number of medical staff in Eastern Taiwan (78.3 staff per 10,000 population) was significantly higher than that in the Taipei region (69.7 staff per 10,000 population) (*p* < 0.001). Taking only permanent residents into account, the number of medical staff in Eastern Taiwan (89.1 staff per 10,000 population) was still significantly greater than that in the Taipei region (66.4 staff per 10,000 population) (*p* < 0.001) and the number of physicians per 10,000 population in Eastern Taiwan (14.3 physicians per 10,000 population) was significantly higher than that in the Taipei region (12.7 physicians per 10,000 population) (*p* < 0.01) (Table 2).

**Figure 3:**
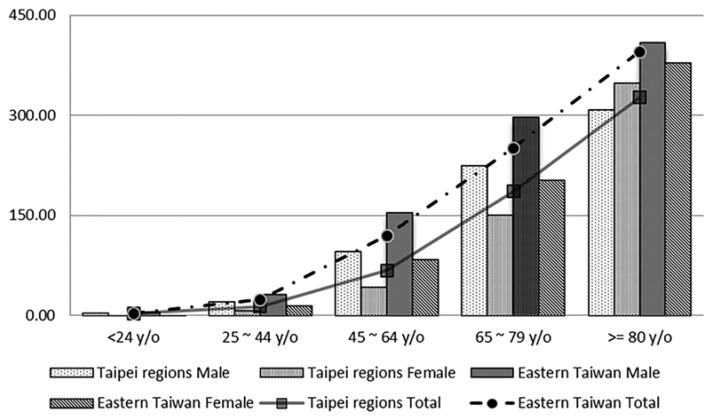
Incidence of sICH (cases per 100,000 population) by age in the Taipei region (urban area) and Eastern Taiwan (rural area).

The total numbers of sICH cases in the Taipei region and Eastern Taiwan were 29,741 (male/female = 62.5%/37.5%) and 4565 cases (male/female = 63.7%/36.3%), respectively. However, the incidence per 100,000 population per year in Eastern Taiwan (71.3 cases per 100,000 population per year) was significantly higher than that in the Taipei region (42.3 cases per 100,000 population per year). There were no significant differences in the mean ages of both the male and female sICH patients between the two areas (Table 3). The incidence of sICH increased with ages; however, the increasing trend of incidence was more significant in Eastern Taiwan than in the Taipei region in both the male and female populations (*p* < 0.001) (Figure 3). 

Because veteran patients are of a relatively low economic status in Taiwan, this study aggregated both low-income patients and veteran patients. The proportion of low-income and veteran sICH patients in Eastern Taiwan (10.6%) did not differ significantly from that in the Taipei region (8.5%). The Charlson Comorbidity Index (CCI) was also calculated in this study, and there was no significant difference in the CCI score between the two areas (Table 3).

Regarding the usage of medical facilities and medical expenditure, the mean hospital length of stay (LOS) in the Taipei region (17.9 days, SD = 15.13) was significantly longer than that in Eastern Taiwan (16.3 days, SD = 13.82) (*p* < 0.001), but there was no significant difference in the intensive care unit (ICU) LOS between the Taipei region (10.2 days, SD = 17.13) and Eastern Taiwan (8.4 days, SD = 10.60). This study also found that there was no significant difference in the proportion of patients with a prolonged ICU stay (35.4% vs. 26.9%), the brain surgical rates (24.9 % vs. 29.3%) and total hospital expenditure (US$4674 vs. US$4873) between the Taipei region and Eastern Taiwan. This study also examined the sub-classifications of hospital expenditure. The sICH patients in the Taipei region (US$253, SD = 212.4, US$277, SD = 380.3 and US$740, SD = 2536.7) had higher medical expenditures than those in Eastern Taiwan (US$235, SD = 202.2, US$225, SD = 313 and US$723, SD = 1358.1) in terms of doctor fees (*p* < 0.001), examination fees (*p* < 0.05) and medication fees (*p* < 0.001) (Table 3).

## 4. Discussion

Big data analytics is a modern method used to increase knowledge; this method of integrating multi-scale large data from different sources will revolutionize the health care system [9,18]. Visualization analysis, a big data analytics tool, can unveil hidden knowledge from big data [19]. The NHIRD (National Health Insurance Research Database) has therefore grown to be a massive and rich database for researchers. When this structured huge database is integrated with other unstructured government open data, it becomes an even more useful big data tool, and researchers are optimistic regarding the discovery of new knowledge in the area of health care that will enable improvement in the quality of health care. This study focused on sICH patients, integrating Taiwan National Health Insurance data and other government open data. The geographical distribution of the sICH patients was demonstrated using the visualization tool of big data analytics, and we found that the incidence of sICH in Eastern Taiwan was significantly higher than that in other regions (Figure 2). This result inspired us to examine the possible reasons behind this finding.

The first possible reason identified in this study that influences the incidence of sICH was the distance barrier. Schoeps et al. [20] found that geographic distance influences the accessibility of health care and affects the survival of children. Rural regions also have disparities in chronic care, surgical intervention abilities and emergency care [21]. This study found that the mean coverage area per hospital in Eastern Taiwan (452.4 km^2^) was almost 20 times that in the Taipei region (24 km^2^). Hypertension would be better controlled to prevent sICH and other associated complications if health care facilities were more accessible [22]. Due to the high density of hospitals, patients with hypertension or other comorbidities that influence sICH in urban areas will have easier access to health care facilities than patients in rural areas. This may explain the higher incidence of sICH in Eastern Taiwan.

Looking at the issue from another perspective, Eastern Taiwan is a longitudinal valley. The East Rift Valley is a long, narrow valley flanked by the Central Mountain Range to the west and the Coastal Mountain Range to the east. It became the barriers of the accessibility of the health care facilities. For example, if a sICH patient in Xiufeng Township, at the northern pole of Hualien County, visited a medical centre in Eastern Taiwan, the journey would be 58.6 km as calculated using Google maps, and would take one hour and 20 min by car. Some stroke patients are unable to reach hospitals soon enough for necessary ultra-early treatment [6]. Although the numbers of medical staff and physicians per 10,000 population did not differ significantly between the two regions, patients in Eastern Taiwan have a lower accessibility to health care facilities due to the distance barrier. The incidence of sICH is therefore increased due to poor care of chronic conditions.

Another reason that influences the incidence of sICH is patient education. Education improves health care-seeking behavior [5]. A low-income status, lower educational status and rural location influence health care accessibility and treatment outcome, [7] and health education could improve patients’ self-care abilities [23]. This study found that the patients in Eastern Taiwan had a lower educational status than those in the Taipei region. For future improvement in the quality of health care, the government should invest more in patient education.

This study had some limitations. First, the Taiwan National Health Insurance data were not real-time data, and patients and conditions could only be identified retrospectively. However, this study presented a feasible healthcare big data model for the integration of administrative data and other government open data. These models can be used to support the government’s medical decision-making. This study also found a significantly higher incidence of sICH in Eastern Taiwan using visualization tools than in other regions.

## 5. Conclusions

This study is a good example of the use of big data analysis by taking advantage of data visualization tools to guide the research direction. Second, the administrative data did not exactly mirror the real clinical data. For example, we may use the patients’ administrative data to calculate what kind of drugs they intake, but how are the status of their medication adherence still remain unclear. This is a significant limitation, as we were unable to calibrate the severity of illness and compare the outcome between groups. Although this study calibrated the severity of illness using the Charlson Comorbidity Index (CCI), we were only able to present the outcome and severities of patients using indirect data. In future studies, we plan to conduct sequential pattern analysis of administrative and other government open data in order to follow-up cases to the final outcome for further evaluation.

## Figures and Tables

**Table 1:** Demographic data of the Taipei region (urban area) and Eastern Taiwan (rural area).

Regions	Taipei Region (Urban Area)	Eastern Taiwan (Rural Area)
Region Demographic Data		
Resident population (average 2001 ~ 2011)	6,396,597	582,108
Male	3,167,119 (49.51%)	305,268 (52.44%)
Female	3,229,478 (50.49%)	276,840 (47.56%)
<24 y/o	2,030,830 (31.75%)	186,927 (32.11%)
25~44 y/o	2,152,713 (33.65%)	184,737 (31.74%)
45~64 y/o	1,631,497 (25.51%)	140,433 (24.12%)
65~79 y/o	448,614 (7.01%)	54,396 (9.34%)
≥80 y/o	132,943 (2.08%)	15,615 (2.68%)
Permanent resident population (2010)	6,709,982	510,980
Area (km^2^)	2324.4	8143.9
Resident population density ***	2751.93	71.48
Permanent resident population density ***	2861.42	62.38
Average income per year (US$) ***	10,112	4200
Total low-income residents (2010) (%) ***	1.3%	3.1%
Smoker (average 2006~2010) (%)	20.84%	23.51%
Educational Status of Residents ***		
Elementary school	12.1%	25.0%
Junior high school	12.3%	16.7%
Senior high school	28.9%	32.7%
University/college	46.7%	25.5%

* *p* < 0.05; ** *p* < 0.01; *** *p* < 0.001.

**Table 2:** Health care facilities in the Taipei region (urban area) and Eastern Taiwan (rural area).

Regions	Taipei Region (Urban Area)	Eastern Taiwan (Rural Area)
Hospital Facilities		
Hospital numbers	97	18
Medical centres	12	1
Regional hospitals	15	3
Mean coverage area per hospital (km^2^)	24	452.4
Total hospital beds	33,570	5588
Total ICU beds	1861	238
Hospital beds per 10,000 population	52.6	96
ICU beds per 10,000 population	2.9	4.1
Hospital beds per 10,000 population (permanent)	50	109.4
ICU beds per 10,000 population (permanent)	2.8	4.7
Medical Staff		
Medical staff	44,556	4555
Physicians	8529	733
Medical staff per 10,000 population ***	69.7	78.3
Physicians per 10,000 population *	13.3	12.6
Medical staff per 10,000 population (permanent) ***	66.4	89.1
Physicians per 10,000 population (permanent) **	12.7	

* p < 0.05; ** *p* < 0.01; *** *p* < 0.001.

**Table 3:** Data for intracranial hemorrhage (sICH) patients in the Taipei region (urban area) and Eastern Taiwan (rural area).

Regions	Taipei Region (Urban Area)	Eastern Taiwan (Rural Area)
Total case number (2001–2011)	29,741	4565
Male	18,597 (62.5%)	2910 (63.5%)
Female	11,144 (37.5%)	1655 (36.3%)
Incidence per 100,000 population per year ***	42.3	71.3
Male	53.4	86.7
Female	31.4	54.3
Mean age	62.4 (16.34)	62.7 (15.58)
Male	60.7 (15.95)	61.6 (15.78)
Female	65.2 (16.59)	64.6 (15.03)
Low-income and veteran patients (%)	2525 (8.5%)	483 (10.6%)
Charlson Comorbidity Index (CCI)		
0 (%)	14,569 (49.0%)	2271 (49.8%)
1 (%)	7678 (25.8%)	1283 (28.1%)
2 (%)	4918 (16.5%)	732 (16.0%)
3 (%)	1512 (5.1%)	162 (3.5%)
≥4 (%)	1064 (3.6%)	117 (2.6%)
Mean hospital length of stay (SD) ***	17.9 (15.13)	16.3 (13.82)
Mean ICU length of stay	10.2 (17.13)	8.4 (10.60)
Prolonged ICU stay	35.4%	26.9%
Surgical intervention rate	24.9%	29.3%
^§^Total hospital expenditure (SD)	4674 (5977.8)	4873 (5433.8)
Doctor fees (SD) ***	253 (212,4)	235 (202.2)
Room fees (SD) *	1442 (1658.6)	1404 (1579.7)
Examination fees (SD) *	277 (380.3)	225 (313.0)
Surgical fees if received surgery (SD)	1333 (1071.1)	1310 (1014.1)
Medications fees (SD) ***	740 (2536.7)	723 (1358.1)

^§^ The amounts in this table are presented in US dollars (US$); * *p* < 0.05; ** *p* < 0.01; *** *p* < 0.001.
